# Diving into a dead-end: asymmetric evolution of diving drives diversity and disparity shifts in waterbirds

**DOI:** 10.1098/rspb.2022.2056

**Published:** 2022-12-21

**Authors:** Joshua Tyler, Jane L. Younger

**Affiliations:** ^1^ Milner Centre for Evolution, Department of Life Sciences, University of Bath, Claverton Down, Bath BA2 7AY, UK; ^2^ Institute for Marine and Antarctic Studies, University of Tasmania, Battery Point, Hobart, Tasmania 7004, Australia

**Keywords:** niche adaptation, convergence, macroevolution, ratchets, Aequorlitornithes

## Abstract

Diving is a relatively uncommon and highly specialized foraging strategy in birds, mostly observed within the Aequorlitornithes (waterbirds) by groups such as penguins, cormorants and alcids. Three key diving techniques are employed within waterbirds: wing-propelled pursuit diving (e.g. penguins), foot-propelled pursuit diving (e.g. cormorants) and plunge diving (e.g. gannets). How many times diving evolved within waterbirds, whether plunge diving is an intermediate state between aerial foraging and submarine diving, and whether the transition to a diving niche is reversible are not known. Here, we elucidate the evolutionary history of diving in waterbirds. We show that diving has been acquired independently at least 14 times within waterbirds, and this acquisition is apparently irreversible, in a striking example of asymmetric evolution. All three modes of diving have evolved independently, with no evidence for plunge diving as an intermediate evolutionary state. Net diversification rates differ significantly between diving versus non-diving lineages, with some diving clades apparently prone to extinction. We find that body mass is evolving under multiple macroevolutionary regimes, with unique optima for each diving type with varying degrees of constraint. Our findings highlight the vulnerability of highly specialized lineages during the ongoing sixth mass extinction.

## 1. Introduction

The emergence of new foraging niches via key innovations can increase both diversity and disparity by releasing taxa from former constraints. Innovations that have opened up new ecological opportunities have often resulted in adaptive radiations, for example, in Darwin's finches in the Galapagos [1,2], cichlid fishes in East African lakes [3,4] and within both placental and marsupial mammals throughout their evolution [5,6]. Yet, there is inherent directionality in niche shifts resulting from key innovations [7,8]. This dichotomy is a key theme in macroevolution. Dollo's law of irreversibility [9], Cope's rule on body size [10] and ratchet mechanisms in macroevolution [11–13] are all examples of asymmetry in evolution, where the trajectories of trait evolution appear irreversible and directional over various time scales, from generations to epochs.

Given the new evolutionary pressures being applied to the world's biodiversity by climate change [14], understanding if highly specialized taxa are evolutionarily ‘trapped’ in their current niches is of great interest. Taxa with adaptive plasticity may be able to ‘weather the storm' of change, while those specialized taxa evolving under macroevolutionary ratchets may face higher risk due to their lower capacity for change. One example is within penguins where this scenario is already playing out, with generalist foraging gentoo penguins faring better than the sympatric krill-specialist chinstraps in terms of population size [15]. Moreover, the success of gentoos has translated into expansions of their range and recently posited speciation events [15–18].

Aequorlitornithes are a group of waterbirds, covering 727 species across 11 avian orders. While the higher-order taxonomic placement of these species has fluctuated in previous phylogenetic studies [19–21], they were recovered as a single monophyletic clade in more recent analyses [22]. They have a near global distribution and use habitats across the continents and oceans [23]. The group includes Charadiiformes (shorebirds, gulls, terns, woodcocks etc. *n* = 369), Ciconiiformes (storks and openbills, *n* = 19), Eurypygiformes (sunbittern and kagu, *n* = 2), Gaviiformes (loons, *n* = 5), Pelecaniformes (herons, pelicans, ibis, bitterns etc. *n* = 106), Phaethontiformes (tropicbirds, *n* = 3), Phoenicopteriformes (flamingos, *n* = 6), Podicipediformes (grebes, *n* = 19), Procellariiformes (albatross, petrels, shearwaters etc. *n* = 128), Sphenisciformes (penguins, *n* = 18) and Suliformes (cormorants, shags, boobies etc. *n* = 52) [22]. Aequorlitornithes represents the largest clade of aquatic species within Aves, with other aquatic species appearing primarily within Anseriformes (waterfowl), Gruiformes (rails, cranes, moorhens etc.) and Coraciiformes (kingfishers) [22,23]. Most of the Aequorlitornithes forage in marine, coastal or freshwater environments, taking a range of prey from insects to fish. Several clades within Aequorlitornithes have evolved the ability to dive to target underwater prey.

Diving is a trait reliant on a suite of highly specialized morphological adaptations [24–27]. As such, diving may represent a key innovation responsible for opening new ecological opportunity, while simultaneously underpinning an evolutionary ratchet dooming diving clades to decline. Diving as a foraging strategy in birds is relatively uncommon, and several studies have focused on the evolution of the strategy in specific taxonomic groups (e.g. penguins [28], Charadriiformes [29], loons and grebes [30], dippers [31], kingfishers [32] and Hesperornithiformes [33]). Many of these groups are found within Aequorlitornithes and several different diving techniques have evolved, including pursuit diving (wing and foot propelled) and plunge diving, with other taxa occupying various non-diving niches.

Here, we used recent high-resolution phylogenies and trait simulation techniques to explore the evolution of diving across all waterbirds. Specifically, we address the following hypotheses: (i) that the evolution of diving in waterbirds is asymmetric and transitions and reversions are rare; (ii) diving represents a key innovation resulting in higher rates of speciation and diversification; and (iii) diving represents a release from former ecological constraints and results in shifts in body mass.

## 2. Results

### (a) Asymmetry of the evolution of diving

To assess whether the evolution of diving in waterbirds is asymmetric, we estimated the number of transitions to/from a diving niche. The 727 species of waterbird were each assigned to one of four diving traits (four-state system): non-diving, plunge diving, pursuit foot diving and pursuit wing diving. These four states were also reduced to a binary system of non-diving versus diving (two-state system). A suite of Markov models of discrete trait evolution were compared, ranging from equal transition rates among all traits to an all rates differ (ARD) model [34,35].

**Figure 1.**
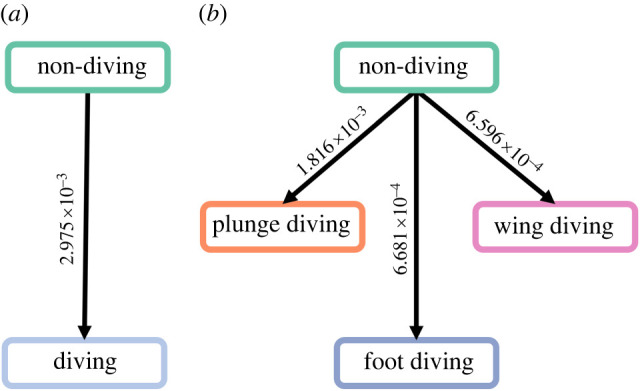
Transition rate models for the evolution of diving. Results from the fitMK models for (*a*) two-state system (no reversion model) and (*b*) four-state system (no reversion model). Values on arrows indicate transition rates. Both best-fit models are asymmetric and unidirectional, with a transition rate of zero for diving to non-diving. (Online version in colour.)

**Figure 2.**
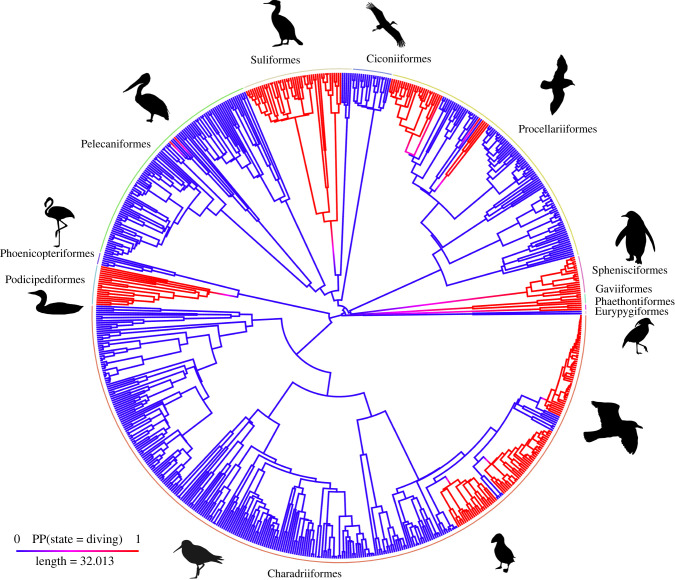
Multiple, convergent acquisitions of diving within Aequorlitornithes. Ancestral state reconstructions for the two-state system: diving (red) versus non-diving (blue). Our analyses recovered an average of 13.6 independent transitions to diving with no reversions, based on 100 simulations. Diving evolved convergently in 8 of the 11 orders within Aequorlitornithes. Avian orders are shown around circumference.

In the two-state system, both the no reversion and ARD models of evolution had high support based on Akaike information criterion (AIC) weight (0.71 and 0.26, table 1) and converged on the same solution whereby the transition rate from diving to non-diving was zero, with a transition rate towards diving of 2.97 × 10^−3^ (figure 1*a*). Our ancestral state reconstructions recovered an average of 14 (95% HPD: 13–15) independent transitions to diving with no reversions (figure 2).

**Table 1.** Mk model fits for the two-state system. Ordered by AIC weight. Transition rate given per million years.

model rank	model name	AIC weight	*Δ*AIC	non-diving to diving rate	diving to non-diving rate
1	NR	0.7075	0	2.97 × 10^−3^	0.00
2	ARD	0.2603	2	2.97 × 10^−3^	0.00
3	ER	0.0323	6.17	2.38 × 10^−3^	2.38 × 10^−3^

In the four-state system, the highest support (AIC weight 0.43; table 2) was for a no reversion all model which set the transition rate from diving states to non-diving and between diving states as zero. The remaining rates for transitions from non-diving to plunge diving, foot diving and wing diving were found to be 1.82 × 10^−3^, 6.68 × 10^−4^ and 6.60 × 10^−4^, respectively (figure 1*b*; electronic supplementary material, A). Under this model, the ancestral state reconstructions found an average of 14 transitions among diving states within waterbirds: eight from non-diving to plunge diving (95% HPD: 8–9); three from non-diving to pursuit foot diving (95% HPD: 3–3) and three from non-diving to pursuit wing diving (95% HPD: 3–4; figure 3 and table 3). The no reversion equal rates model is also well supported (AIC weight 0.37, ΔAIC = 0.30). Both of these models do not allow reversions back to non-diving, but the latter model allows for transitions between diving forms. Under this second model, the ancestral state reconstructions find that the average number of transitions from non-diving to pursuit foot diving is unchanged (3; 95% HPD: 2–4), as is the number from non-diving to pursuit wing diving (3; 95% HPD: 2–4). The only difference in transitions between the two models is a reduction in non-diving to plunge diving transitions from 8 to 7 (95% HPD: 7–9), and the introduction of a single transition from pursuit foot diving to plunge diving (95% HPD: 0–2). The total number of transitions remains the same between the two models.

**Table 2.** Mk model fits for the four-state system. Ordered by AIC weight. Transition rates provided in electronic supplementary material, A.

model rank	model name	AIC weight	*Δ*AIC	log-likelihood
1	NR ALL	0.4306	0	−82.94
2	NR ER	0.3698	0.3045	−85.09
3	ER	0.1386	2.2668	−86.07
4	NR SYM	0.0430	4.6069	−82.24
5	SYM	0.0153	6.6719	−83.27
6	NR ARD	0.0025	10.2296	−82.05
7	ARD	0.0001	16.2296	−82.05

**Figure 3.**
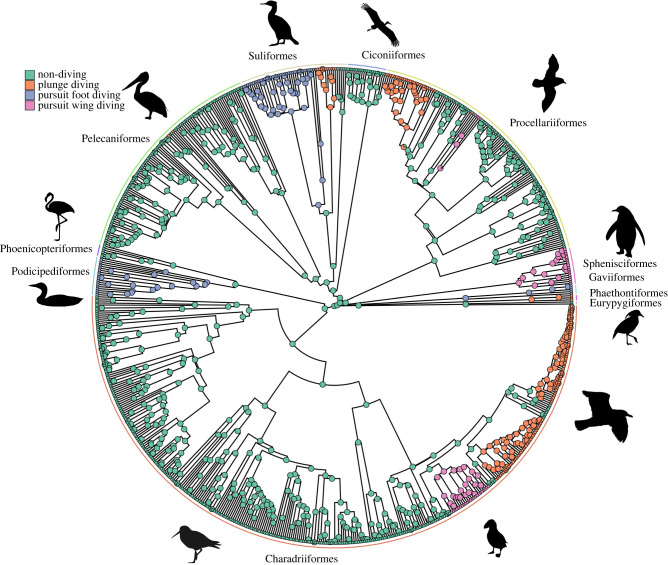
The evolution of diving niche in waterbirds. Ancestral state reconstruction for the four-state system (non-diving, foot diving, wing diving and plunge diving) using the no reversions all model. Pie charts on the internal nodes represent the proportion of assignment to each state over the 100 simulations. Our analyses recovered an average of 8, 3 and 3 independent transitions to plunge diving, pursuit foot diving and pursuit wing diving, respectively, based on 100 simulations. Avian orders shown around circumference.

**Table 3.** The average number of transitions between foraging niches in the four-state system. Table reads row to column. Based on 100 SIMMAP simulations.

from	non-diving	plunge diving	foot diving	wing diving
non-diving	n.a.	8.25	3.00	3.06
plunge diving	0	n.a.	0	0
foot diving	0	0	n.a.	0
wing diving	0	0	0	n.a.

### (b) Net diversification rates are correlated with foraging traits

**Figure 4.**
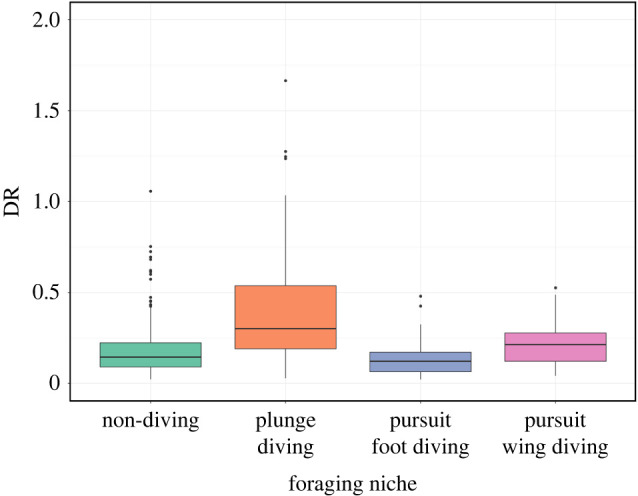
Speciation rate (DR) comparisons between diving niches. Diving taxa had a slightly higher speciation rates on average compared to non-diving taxa, but all phylogenetically corrected comparisons were statistically non-significant. *y*-axis is truncated and 10 species with DR > 2 are not shown for clarity (all within plunge diving niche). Taxon distribution: non-diving: 491, plunge diving: 130, pursuit foot diving: 61 and pursuit wing diving: 45. Figure 3 and electronic supplementary material, B for foraging niche classifications. (Online version in colour.)

Speciation and diversification rates across the waterbird phylogeny were calculated to determine whether diving represents a key innovation facilitating a higher speciation or diversification rate. Tip rates (DR) were used as they provide a species-level metric that can be compared across the tree [19,36]. We found that diving taxa have higher average speciation rates (DR) than non-diving taxa (diving: mean = 0.598, median = 0.245; non-diving: mean = 0.183, median = 0.145). This difference was significant based on a standard ANOVA, but non-significant using a phyloANOVA method (*p* = 4.97 × 10^−12^ and *p* = 0.219, *η*^2^ = 0.06), attributable to the clustered nature of the diving on the phylogeny within certain orders. Only the standard ANOVA across the four-state system was highly significant (*p* = 4.97 × 10^−12^, *η*^2^ = 0.14) with the phylogenetic ANOVA and pairwise *t*-tests producing non-significant results (figure 4). When comparing speciation between the four dietary categories (plant/seed, omnivore, invertebrate and vertebrate/fish/scavenging), there were no significant differences in DR using a phyloANOVA method with a Holm *post hoc* correction (*p* = 1.00 for all corrected pairwise comparisons).

**Figure 5.**
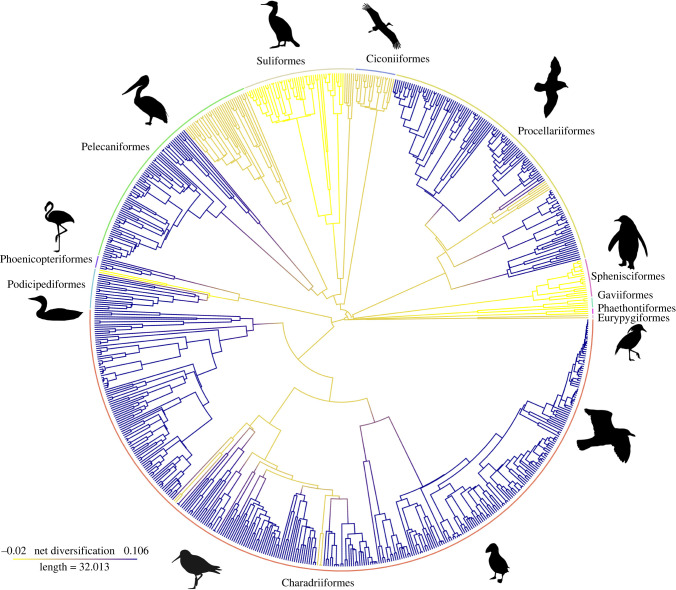
Net Diversification shifts between diving niches. Based on the HiSSE Full model (allowing for two-defined states—non-diving (0) and diving (1); and two hidden states—A and B), there are four regimes with an associated net diversification rate (given as speciation rate minus extinction rate). The non-diving regimes both have positive rates (0A: 1.06 × 10^−1^ and 0B: 4.69 × 10^−3^) while the diving regimes have a positive and a negative rate (1A: 1.06 × 10^−1^ and1B: −1.97 × 10^−2^). Avian orders shown around circumference.

Hidden state speciation and extinction (HiSSE) models allow for transitions between niches and net diversification rates within niches to be jointly estimated, providing a further method to understand the role of niche within speciation and extinction [37,38]. Simulations find strong support for the HiSSE Full model (speciation rates and extinction rates depend on both the diving character and a hidden character) (AIC weights ∼ 1) (table 4). Of the four regimes recovered, both non-diving regimes have positive net diversification (0A: 1.06 × 10^−1^ and 0B: 4.69 × 10^−3^) while the diving regimes have a positive and a negative rate (1A: 1.06 × 10^−1^ and1B: −1.97 × 10^−2^) (figure 5; see electronic supplementary material, B for hidden state assignment probabilities).

**Table 4.** HiSSE model results. The number of regimes corresponds to the sum of given and hidden states (i.e. for HiSSE full, there are two diving states, 0 and 1, and two hidden states, A and B).

model rank	model name	no. of regimes	AIC weight	*Δ*AIC
1	HiSSE full	4	∼1	0
2	HiSSE null	4	0	178.6
3	BiSSE null	2	0	246.2
4	HiSSE non-diving	3	0	568.8
5	HiSSE diving	3	0	1579.3
6	BiSSE	2	0	2718.3
7	dull null	1	0	2836.5

### (c) Shifts in morphology following the evolution of diving

**Figure 6.**
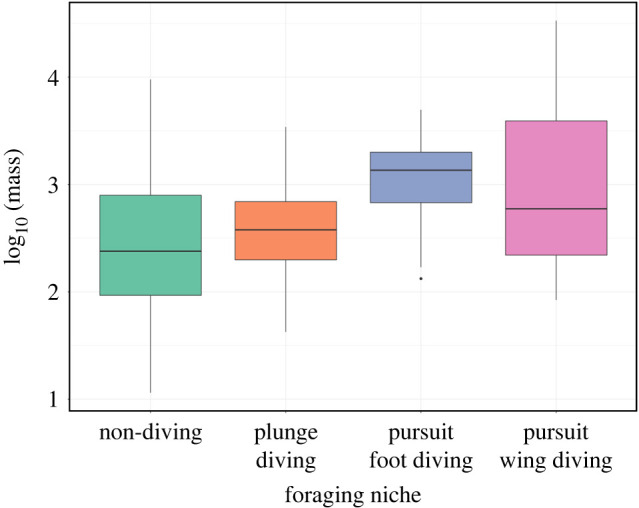
Body size comparisons between diving niches. Values shown are log_10_(mass). Taxon distribution: non-diving: 491, plunge diving: 130, pursuit foot diving: 61 and pursuit wing diving: 45. Figure 3 and electronic supplementary material, B for foraging niche classifications. (Online version in colour.)

Body mass is a key morphological character and has been shown to dominate disparity signals in birds [39]. In the two-state system, the standard ANOVA, not phylogenetic ANOVA, testing finds highly significant differences in mass distributions (*p* = 1.85 × 10^−10^ and *p* = 0.219, *η*^2^ = 0.05) with diving birds occupying a heavier range. In the four-state system, pairwise *t*-tests find significant differences between several states (*p*-values: non-diving—foot diving: 4.3 × 10^−12^, non-diving—wing diving: 1.5 × 10^−6^, plunge diving—foot diving: 1.9 × 10^−6^, plunge diving—wing diving: 1.6 × 10^−3^) but non-significant results when phylogeny is considered (figure 6).

We considered the evolution of body mass on the phylogeny using multiple diffusion models of continuous trait evolution (covering both Brownian motion and Ornstein–Uhlenbeck processes), grouping taxa according to both the two- and four-state systems [40–44]. Using AIC weights, the best-fitting models for body mass evolution is OUMVA using the four-state system (AIC weight = 0.98). The OUMVA model fits differing optima, alpha and variance parameters to each regime. These results correspond to wing diving birds having the heaviest body mass optima (*θ* = 4.58) followed by foot divers (*θ* = 3.00), with non-divers and plunge-divers having similarly small optima (*θ* = 2.76 and 2.74, respectively). Both the alpha and variance parameters describe movement towards and around the trait optima. Plunge divers have the largest alpha and smallest variance of the four regimes (0.103 and 4.48 × 10^−6^) with others having more intermediate values: non-diving: 0.025 and 0.024; foot diving: 0.044 and 0.005; and wing diving: 0.015 and 0.019. The alpha parameter can be converted into the phylogenetic half-life which indicates the strength of decay towards the optima. Wing divers have the longest half-life (46.1 Ma), compared to non-diving (27.7 Ma), foot diving (15.8 Ma) and plunge diving (6.71 Ma) (see electronic supplementary material, C for full results). Simpler models where only certain parameters were free had significantly lower support.

## 3. Discussion

Here, we show that the evolution of diving is irreversible in waterbirds. Transitions from non-diving to diving have occurred multiple times within modern Aequorlitornithes, but according to our analyses, this is always a unidirectional acquisition. The evolution of diving therefore represents a macroevolutionary ratchet (i.e. repeated evolution towards a specialism from which reversion is not predicted). These ratchet patterns have been repeatedly found in the evolution of mammalian carnivores [11,12,45,46], but here we develop one of the first large-scale examples in birds, using the case of diving in waterbirds.

Furthermore, based on the most highly supported model of evolution (no reversions all), we find that plunge diving is most likely not a required intermediate state between ariel and submarine flight, but rather all three modes of diving have evolved independently and convergently across the phylogeny. Diving has evolved in 8 of the 11 avian orders within Aequorlitornithes, with 14 unique transition events. Five of these transitions occurred on stems leading to orders (Podicipediformes, Suliformes, Sphenisciformes, Gaviiformes and Phaethontifomes), while the other nine transitions are deeply nested within orders (twice within Pelecaniformes, four times within Procellariiformes and three times within Charadriiformes). In the alternate model of evolution (no reversions ER), all but 1 of the 14 transitions are unambiguously an acquisition of diving by a non-diving ancestor. Our discrete trait modelling predicts a single transition between diving niches (pursuit foot diving to plunge diving, within the Suliformes); however, this transition is only predicted in 87% of simulations, with the remaining 13% favouring a non-diving ancestor. Given the paucity of orders where multiple diving niches have evolved and the lack of other transitions among diving niches, the result within Suliformes should be viewed with some caution.

We find evidence that the evolution of diving influences net diversification rather than speciation rates within Aequorlitornithes, yet diving does not appear to be a key innovation promoting speciation via ecological release [7,8]. This may be because, while diving provides birds with access to a new environment, the aquatic niche is already partially occupied (e.g. by marine mammal, reptile and fish lineages [47,48]).

Our results indicate that some diving clades are experiencing negative net diversification (i.e. extinction rates larger than speciation rates). Of the 236 diving taxa included in the analysis, 75 (32%) were assigned to this negative diversification regime, including 72% of pursuit foot divers, 40% of pursuit wing divers and 10% of plunge divers. The results from our hidden state models support the idea that diving is a partial driver of diversification shifts, with the HiSSE model indicating that there are other traits underpinning diversification regimes within waterbirds. It appears that the interaction between the diving state and the hidden state is the key driver rather than diving alone. This is reinforced by all taxa in hidden state A, irrespective of foraging strategy, having an equivalent diversification rate, which effectively reduces the four regimes to three, indicating that the hidden state is an important factor. Further studies incorporating wider behavioural, ecological or environmental trait data may help elucidate these hidden drivers of speciation shifts in Aequorlitornithes.

Our analysis of phenotypic patterns shows that pursuit diving taxa are evolving towards larger body masses, with different optima for each diving strategy, while plunge diving and non-diving taxa have a similar smaller optimum. This is consistent with our understanding of diving physiology, in which larger body sizes seem to be selected for in diving birds and mammals, allowing for longer dive durations and deeper depths due to reduced metabolic rates, greater oxygen storage and greater insulation [48–50]. Moreover, our models show considerably more constraint on plunge diving taxa compared to non-diving and pursuit diving lineages, in terms of both the variance in body size and strength of selection towards the optimum. Essentially, the selective pressure for birds that rely on plunge diving for foraging to have body sizes around the optima is more intense than the selective pressure on size for other strategies, which may be connected with the specific biomechanical requirements for plunge diving [26,32,51].

The possibility that diving taxa may be evolutionarily ‘trapped’ in their current niches does not bode well for the long-term survival of these lineages. According to the IUCN Red List of Threatened Species [14], 156 (21.5%) of the 727 Aequorlitornithes species are already listed as either vulnerable, endangered or critically endangered. When considering diving taxa experiencing negative diversification specifically, that proportion rises to 32% (24 of 75 taxa). It remains to be seen whether diving specialists will be able shift their foraging niches in response to emerging evolutionary pressures, given the unidirectionality in the evolution of diving we have shown here.

## 4. Conclusion

The evolution of diving in Aequorlitornithes has occurred multiple times independently across the clade and is accompanied by a shift in morphological optima towards heavier body masses for pursuit divers and an increase in constraint on the range of body masses occupied by plunge divers especially. The shifts in discrete and continuous traits reflect macroevolutionary ratchets, with an inherent directionality and lack of reversals. These patterns, alongside the convergence on strategy and form, point towards evolutionary processes that favour the exploration of new forms and functions rather than a return to an ancestral state. However, these are associated with changes in net diversification rate and point towards a pattern of species filling moving niches in an adaptive landscape rather than exploring all options. With many diving taxa at risk as marine prey resources decline, this result reiterates the need to protect waterbirds globally.

## 5. Methods

### (a) Phylogenetic framework

The analyses are based on a composite tree of all birds with 100% species coverage, following the process implemented in Cooney *et al*. [52], in which the family level genomic backbone of Prum *et al*. [22] and the within-family topologies of the maximum clade credibility tree of the Hackett backbone [19,20] are combined producing a younger branching topology than previous hypotheses [19–21]. This composite tree was used as it returns a monophyletic clade of waterbirds, *Aequorlitornithes*, comprising 727 species. The composite tree was trimmed to this clade using the *extract.clade* function in *ape* [53], which was then used in subsequent analyses.

### (b) Trait assignments and covariates

Taxa were assigned to a broad foraging niche (non-diving or diving, herein the two-state system) and a detailed foraging niche (non-diving, plunge diving, pursuit foot diving or pursuit wing diving, herein the four-state system). These assignments represent the foraging strategy primarily used by each species and were based on the foraging niches provided in Pigot *et al.* [39]. Here, we take any species listed as using the ‘aquatic plunge' or ‘aquatic dive' foraging niche to be diving in the two-state system. These were then classified into plunge diving, pursuit foot diving and pursuit wing diving in the four-state system. Cases where small numbers of species were listed as ‘aquatic surface' or ‘aquatic aerial' within primarily ‘aquatic plunge' clades were checked individually in Birds of the World [23] to determine if plunge diving was also used by these taxa, and those taxa that fully submerge from plunge diving were recategorized. Any ‘Generalists' of the families Laridae & Procellaridae that fully submerge from plunging were also re-assigned to the plunge diving category. All assignments can be found in electronic supplementary material, B which includes the two-state, four-state and original Pigot foraging niches. IUCN extinction risk categories were obtained from the IUCN Redlist [14], in order to investigate correlations with extinction risk. Primary dietary categorization (plant/seed, omnivore, invertebrate and vertebrate/fish/scavenging) and body mass (log_10_ scaled from original measurement in grams) for each taxon were taken from the EltonTraits 1.0 database [54].

### (c) Model support using the Akaike information criterion

To determine the best model from each model set in the subsequent elements of analysis, we use the Akaike information criterion in order to rank models based on fit [55]. The AIC score is calculated as

AIC= −2ln⁡(L)+2K,

where *L* is the likelihood, given as the probability of the data given the model, and *K* is the number of free parameters.

To measure the relative support for each model *i*, we calculated the ΔAIC value

$$\Delta \mathrm{AIC}={\mathrm{AIC}}_{i}-{\mathrm{AIC}}_{\min},$$

where AIC_min_ is the smallest AIC score. Models that have a ΔAIC of less than 2 should be considered as having substantial support, those between 2 and 7 have minimal support and those greater than 7 have no support. All models with ΔAIC < 2 are reported and interpreted.

We finally calculated the AIC weight based on the AIC scores

$$w_{i}=\frac{\exp(-1/2\Delta {\mathrm{AIC}}_{i})}{\sum_{\;j=1}^{J}\exp(-1/2\Delta {\mathrm{AIC}}_{j})},$$

where *J* is the number of models being assessed. AIC weights sum to 1 and models with higher support have larger values.

### (d) Ancestral states of foraging niche

We estimated ancestral states of foraging niche using stochastic trait mapping as implemented in *phytools* in R [34,35,56]. To ascertain the best transition rate model, we used the fitMK function within *phytools* for a range of Markov models with differing patterns of discrete trait evolution. For the two-state system, three models were compared: ‘equal rates' (where the transition rate between the two states is identical, ‘ER'), ‘ARD' (where the transition rate can vary dependent on direction, ‘ARD') and ‘no reversion' (where the transition rate from diving to non-diving is explicitly set at zero).

Seven models were compared for the evolution of the four-state system; three allowed transitions among all four states (equal rates, symmetric, ARD); a further three set the transition rate from any diving state to non-diving as zero (no reversion equal rates, no reversion symmetric, no reversion ARD); and a final model (no reversions all) where only transitions from non-diving to the diving states were allowed.

The predictive performance of the models was assessed using AIC weights. The best performing models for the two-state and four-state systems were then simulated using the *make.simmap* function in *phytools* with 100 replications. These trait maps were then summarized to generate a posterior estimate of the foraging niche state at each node, and to estimate the mean number of transitions between each state.

### (e) Influence of traits on speciation rate

Speciation rates (expected number of speciation events in the next time unit, in this case millions of years) were calculated for each taxon (*i*) on the phylogeny using tip-associated rates with the following equation:

$${\mathrm{DR}}_{i}={\left(\overset{N_{i}}{\underset{\;j=1}{\sum }}l_{j}\frac{1}{2^{\;j-1}}\right)}^{-1},$$

where *N_i_* = number of edges on path from species *i* to the root and *l_j_* = length of edge *j* [19,36]. This metric represents the inverse of its mean equal splits measure for each species *i,* which is a measure of the expected waiting time per-lineage before another speciation event occurs. The DR statistic was chosen as the primary measure of speciation given its state independence, model-free nature and ease for comparison across many categorizations [36].

To examine the correlation of foraging niche and diet with speciation rate (DR), we carried out a phylogenetic ANOVA test (phylANOVA in *phytools*) with *post hoc* Holm correction for multiple comparisons and eta squared (*η*^2^) measures for effect size.

### (f) Influence of traits on net diversification rate

To test trait-dependence of the net diversification rate across the phylogeny, we applied binary state speciation and extinction (BiSSE [37]) and HiSSE ([38]) methods. The BiSSE framework is a model-based approach to estimate the influence of a single binary trait on diversification rate, whereas the HiSSE framework is a BiSSE extension that can also account for hidden states (i.e. unmeasured traits). We modelled seven scenarios, based on the two-state (diving versus non-diving) system: (i) a dull null where net diversification rate is equal across the tree; (ii) a BiSSE model where the net diversification rate is dependent solely on the diving state; (iii) a model where the net diversification rate is only dependent on the hidden state (a null model for the BiSSE), (iv) a HiSSE model including the diving state and a hidden state where net diversification is dependent on both states (i.e. four regimes); (v) a HiSSE model including the diving state and a hidden state only for non-diving (i.e. three regimes); (vi) a HiSSE model including the diving state and a hidden state only for diving (i.e. three regimes) and (vii) a null version of model 4 where the parameters are estimated only for hidden states (i.e. four regimes). In all models, we allowed both turnover and extinction fraction to be estimated per regime and set all transitions between hidden states to equal probabilities. The model fits were assessed using AIC weights. State assignment probabilities for the best-fitting model are given in electronic supplementary material, C for each taxon.

### (g) Modelling continuous trait evolution

Body mass distribution comparisons with foraging strategy used a phylogenetic ANOVA test (phylANOVA in *phytools*) with *post hoc* Holm correction for multiple comparisons and eta squared (*η*^2^) measures for effect size.

To test if the tempo and mode of body size evolution was influenced by diving, we fit a series of macroevolutionary diffusion models to the comparative body mass dataset mapped onto the SIMMAP niche trees based on the ancestral state reconstructions for both the two- and four-state systems [34,35,40–42]. In total, 14 models were fit using the *fitContinuous* and *OUwie* functions from the *GEIGER* and *OUwie* R packages [43,44]: four regime-independent models (BM1, OU1, EB and Trend) and five regime-dependent models (BMS, OUM, OUMA, OUMV and OUMVA) which were run for both the two-state and four-state systems. Relative support for each model was assessed using the associated AIC weights Phylogenetic half-life was calculated by dividing log(2) by the calculated alpha parameter and indicated the amount of time for the trait value to decay half the distance towards the trait optima [43]. In order to test if flightless taxa were having an impact on the results, we also ran the five regime-dependent models again with flightless taxa in their own regime (i.e. non-diving, diving and flightless). Included taxa were all species within family Spheniscidae, *Nannopterum harrisi*, *Podiceps taczanowskii* and *Rollandia microptera*.

## Data Availability

All data available either in original publications cited or in the electronic supplementary material including R code used for analysis. Package versions: R (4.1.2), OUwie (2.6), hisse (2.1.6), geiger (2.0.7), phytools (1.1–7), ape (5.6–2), ggplot2 (3.3.5) and tidyverse (1.3.1). The data are provided in electronic supplementary material [57].
